# Novel stress phonotactics are learnable by English speakers: Novel tone phonotactics are not

**DOI:** 10.3758/s13421-019-01000-9

**Published:** 2019-12-26

**Authors:** Yuan Bian, Gary S. Dell

**Affiliations:** 1 Massachusetts Institute of Technology, Cambridge, MA USA; 2 University of Illinois, Urbana-Champaign, IL USA

**Keywords:** Implicit learning, Language production, Phonotactic learning, Speech error

## Abstract

Speech errors are sensitive to newly learned phonotactic constraints. For example, if speakers produce strings of syllables in which */f/ is an onset if the vowel is* /æ/*, but a coda if the vowel is* /*I*/, their slips will respect that constraint after a period of sleep. Constraints in which the contextual factor is nonlinguistic, however, do not appear to be learnable by this method—for example, */f/ is an onset if the speech rate is fast, but* /*f*/ *is a coda if the speech rate is slow*. The present study demonstrated that adult English speakers can learn (after a sleep period) constraints based on stress (e.g., /*f*/ *is an onset if the syllable is stressed, but* /*f*/ *is a coda if the syllable is unstressed*), but cannot learn analogous constraints based on tone (e.g., /*f*/ *is an onset if the tone is rising, but* /*f*/ *is a coda if the tone is falling*). The results are consistent with the fact that, in English, stress is a relevant lexical phonological property (e.g., “INsight” and “inCITE” are different words), but tone is not (e.g., “yes!” and “yes?” are the same word, despite their different pragmatic functions). The results provide useful constraints on how consolidation effects in learning may interact with early learning experiences.

One’s native language is learned in childhood. At the same time, it is acknowledged that the mature language processing and production systems continue to reflect linguistic experience throughout life. Learning new vocabulary is an uncontroversial example of continuing acquisition. Psycholinguistic findings have also shown that linguistic structures or rules are changeable. For example, syntactic priming (Bock, 1986; see Dell & Ferreira, 2016) in adult speakers has been attributed to an ongoing implicit learning process (e.g., Chang, Dell, & Bock, 2006).

Our work addresses the ability of the mature language production system to adapt to novel *phonotactic* patterns. Each language has its own *phonotactics*—that is, a set of rules that constrain possible phoneme sequences. For example, in English, /ŋ/ (*ng* sound) can only be at the coda (i.e., ending) position of a syllable, as in “sing.” But in Vietnamese, /ŋ/ can be an onset (i.e., beginning), as in /ŋɪj˧˧/ (meaning: “to doubt”). Phonotactic knowledge is acquired from linguistic experience during infancy and childhood, and it systematically influences both language perception and production (see Warker & Dell, 2006, for a review).

A number of studies have demonstrated that the effects of phonotactic knowledge on linguistic performance can be changed by experience in the laboratory. This experience affects both perception (e.g., Onishi, Chambers, & Fisher, 2002) and production (e.g., Dell, Reed, Adams, & Meyer, 2000) and occurs in participants of all ages (e.g., Chambers, Onishi, & Fisher, 2003). In this article, we demonstrate such changes in the production systems of adult speakers, and specifically, that the changeability of the mature system is correlated with prior linguistic experience: Native English speakers can learn new rules about how syllabic stress constrains what can be an onset or coda, but they cannot learn analogous rules regarding syllabic tone, even though they can easily produce all of the relevant syllables. This dissociation may arise from the fact that stress, but not tone, distinguishes English lexical items.

Knowledge of phonotactics is implicit, or unconscious. This knowledge, however, is revealed in speech errors (or slips), through the *phonotactic regularity effect*—that is, the tendency for slips to obey the language’s phonotactics (Fromkin, 1971). For example, “list” in English may slip to “tist,” but not to “tlist.” Our studies created an analogue to the phonotactic regularity effect in the laboratory, thus using slips to measure the acquisition of sensitivity to the novel phonotactics exhibited by the syllables that participants experience.

In the original study of this sort, Dell et al. (2000) presented participants with sequences of four CVC syllables containing eight different consonants (always *h*, *ŋ*, *f*, *s*, *m*, *n*, *k*, *g*) and a single vowel. An example sequence is “hes feng mek neg.” The participants simply had to say the syllables aloud in time with a metronome. The consonants in the sequences exhibited three types of constraint:

1. Language-wide constraint: /h/ must be an onset and /ŋ/ must be a coda. Slips involving these consonants were expected to follow English phonotactics; that is, /ŋ/, if it slipped, would always slip to coda position. Consonants subject to this constraint are referred to as *language-restricted consonants*.
2. Experiment-wide constraint: /f/ must be an onset and /s/ must be a coda. This is not generally true for English, just for the experiment. The extent to which slips of /f/ and /s/ obeyed the constraint (e.g., /f/ slips to onset instead of coda position) was a measure of learning of the experiment-wide constraint. Consonants subject to this constraint are referred to as *experiment-restricted consonants*.
3. Experimentally unrestricted consonants: /k, ɡ, m, n/ were not restricted, appearing as both onsets and codas. Errors involving consonants in this category were used as a baseline to compare to the slips involving consonants subject to experiment-wide constraints. Consonants subject to this constraint are referred to as *unrestricted consonants*.

The speech errors that occurred in the experiment were classified according to whether the slipping consonant moved “legally.” For consonants subject to language-wide and experiment-wide constraints, a legal slip obeys the constraint (e.g., onset /h/ or onset /f/ moves to another onset position) and an illegal one (onset /h/ or onset /f/ moves to a coda position) does not. The proportions of slips from these conditions that are legal can be compared to a baseline by examining slips of unrestricted consonants, for example, slips of /k/. Within a sequence, the position of each unrestricted consonant—for example, /k/—is “restricted,” but just for that sequence. This is because each consonant appears just once in each sequence. So, if a /k/ was an onset in the current sequence, if it slipped to another onset position, it was “legally” following the constraint that /k/ is an onset within that sequence. If it slipped instead to a coda position, the slip was “illegal.” For a slip of /k/ in another sequence in which /k/ was a coda, a slip to a coda position would be legal, and a slip to an onset would be illegal. Thus, although the positions of unrestricted consonants and the corresponding implications for slip legality differ for different sequences, the overall proportion of legal slips for unrestricted consonants forms an unbiased baseline that can be compared to the legality proportion of slips of restricted consonants. Dell et al. (2000) found, as expected, that slips of language-restricted consonants were legal 100% of the time. Crucially, they found that experiment-restricted consonant slips were legal 98% of the time, far greater than the baseline of 68% legality for slips of unrestricted consonants.^1^ The manifestation of learning the artificial phonotactic constraints was shown in a higher legality for the experiment-restricted consonant slips than for the unrestricted consonants (this will be referred as “excess legality”). Although Dell et al.’s (2000) experiment was a four-day study, the high rate of legality for experiment-restricted slips was achieved very quickly. The rate on Day 1 was 98%, the same as the mean across all four days. This large and rapid learning effect has been replicated many times (e.g., Goldrick, 2004; Goldrick & Larson, 2008; Kittredge & Dell, 2016; Taylor & Houghton, 2005; Warker & Dell, 2015; see Anderson & Dell, 2018, for a meta-analysis).

When this speech error technique is used with other kinds of constraints, it has been found that not all constraints are learnable and not all learnable constraints are equally easy to learn. The constraints used by Dell et al. (2000) have been called *first-order*. These are unconditioned constraints that certain consonants must occur in certain positions (e.g., /f/ must be an onset, regardless of context). *Second-order* constraints, on the other hand, depend on context. For example, */f/ must be an onset and /s/ must be a coda if /ɪ/ is the vowel in the syllable, but /f/ must be a coda and /s/ must be an onset if /*æ*/ is the vowel*. That is, the positions of /f/ and /s/ depend on some property of the syllable, in this case, the vowel.^2^ As we mentioned above, Dell et al. (2000) found that first-order constraints are fully learned within one experimental session. However, using the same experimental paradigm, Warker and Dell (2006) found that second-order constraints with a vowel contingency do not influence speech errors until a second session on the next day. Anderson and Dell (2018) reviewed eight studies involving a second-order vowel contingency, all of which replicated the finding of no effect on Day 1 (mean excess legality = – 1% compared to baseline), and a reliable effect on Day 2 (7%–24% excess legality). Gaskell et al. (2014) specifically showed that sleep, as opposed to a time-matched awake period, is needed for this second-order learning to be revealed.

Warker, Dell, Whalen, and Gereg (2008) discovered a second-order constraint that could not be learned, even after four days of training. In that study, the constraining context was the rate that each trial sequence was produced, either 2.67 syllables/s (“fast”) or 1.87 syllables/s (“slow”)—for example, /*f*/ *must be an onset and* /*s*/ *must be a coda if the speech rate is fast, but* /*f*/ *must be a coda and* /*s*/ *must be an onset if the rate is slow*. Although the difference in rate was a salient feature of the speakers’ productions and had a very large effect on the number of slips, the slips failed to follow the rule. Warker et al. proposed that extralinguistic features such as speech rate are not present in the production component that represents the positions of speech sounds in syllables. That could be because such features were never present in that production component or because English speakers have learned that they do not constrain English phonotactics, and hence they are excluded as a result of experience. We shall expand on this notion of “extralinguistic” below.

To sum up, the studies of phonotactic learning using this speech-error method have shown three kinds of results: quick learning, learning after a sleep period, and failure to learn. Warker and Dell (2006) developed a connectionist production model that could learn phonotactic patterns, and Dell, Kelley, Bian, and Holmes (2019) situated this model within the Levelt, Roelofs, and Meyer (1999) model of production, resulting in a framework for explaining these effects. Below, we summarize this framework (see Dell et al., 2019, for an expanded discussion).

The Levelt et al. model distinguishes between phonological encoding, in which retrieved word-form information is assembled into syllables (syllabification), and later phonetic encoding processes. During phonological encoding, segments are assigned to syllable positions based on segmental and metrical information retrieved from the lexicon and on phonotactic knowledge. A version of the model for tone languages assumes that retrieved lexical tones, instead of stress-related metrical patterns, participate in this process (e.g., O’Seaghdha, Chen, & Chen, 2010). To explain phonotactic learning effects on speech errors, we must assume that the process of placing segments in syllable positions can be changed through experience. Warker and Dell (2006) used a three-layer connectionist model to explain this adaptation. In this model, an input layer, an output layer, and a layer of hidden units mapped the input onto the output. The input to the model included in principle all the relevant lexical information (e.g., segments, morphemic structure, metrical structure), and the model output was the assignment of segments to syllables and syllable positions (e.g., onset or coda for consonants). The model was trained by producing simplified English syllables, allowing it to learn, for example, that English /h/ must be an onset. The training involved changing the model’s connection strengths, or “weights,” according to the learning algorithm known as backpropagation (see McClelland, Rumelhart, & PDP Research Group, 1987, for details). A connectionist model with hidden units develops functional representations that mediate between input and output by changing the weights to and from hidden units. To learn a second-order rule about how a vowel constrains whether a consonant is an onset or a coda, one or more hidden units have to develop connection weights, so that those units are activated nonlinearly by both the vowel and the consonant. In that way, these hidden units come to represent the conjunction of input features, or functionally, to represent the vowels in the context of consonants (e.g., /f/ in the context of /ɪ/). After training, the model’s slips obeyed English phonotactics. The model was also able to explain the rapid learning of new first-order rules (e.g., /f/ only occurs in onset position), because its input includes the relevant segments. For example, /f/ is a possible input because /f/ is a segment that distinguishes many English lexical items from other items. The model also correctly failed to learn second-order rules in which the contingent factor was speech rate. This occurred for the simple reason that a speech rate value is not a possible input to the model. It is not an input because speech rate does not distinguish lexical items from one another, and hence this feature is not used during phonological encoding. Rather, speech rate impacts production from outside of phonological encoding (e.g., as proposed in MacKay, 1982), and presumably at later stage in the Levelt et al. model.

The Warker–Dell adaptive model of syllabification cannot, however, account for the fact that second-order constraints, such as that /f/ is an onset and /s/ is a coda for one vowel, but the reverse for another vowel, are learned only after a consolidation period. Rather, the model quickly learns these second-order patterns, because the relevant inputs (e.g., /f/, /s/, and the two vowels) are present. Dell et al. (2019) explained the need for consolidation for these kinds of constraints by noting that second-order constraints require people to have functional representations of segmental conjunctions that can represent the context dependency (e.g., /f/ in the context of the vowel /ӕ/). These functional representations correspond in the model to appropriate sets of weights involving the hidden units connected to the relevant inputs (e.g., /f/ and /ӕ/). Dell et al. (2019) assumed that a sleep period might be required to make such representations functional.

Why are the representations of the relevant conjunctions not immediately functional? After all, their inputs (e.g., /f/ and /ӕ/) are clearly part of English phonology. Dell et al. (2019) proposed that these representations have been “backgrounded” as a result of experience with English (see Dell et al., 2019, for details). During childhood, representations of the conjunctions are present (such as /f/ in the context of /æ/) and are used in syllabification during speaking. In the context of the model, these representations inhabit particular hidden units. Dell et al. (2019) proposed that, over time, hidden units that are not actively contributing to syllabification decisions become backgrounded; they function like other hidden units, but they are not able to rapidly change their connection weights. If it is later found that these units are needed, they can be restored after a period of time (or sleep). These assumptions can explain why slips do not follow the second-order vowel contingent rules until the second day.

In English, vowels play a major role during syllabification, but for the most part they do not determine whether consonants or clusters can be onsets or codas (see Dell et al., 2019). Thus, representations of vowel–consonant conjunctions that would help in learning a new second-order vowel-contingent rule would be backgrounded, and hence, any such learning would be not be revealed until the second day. This proposal is also consistent with two examples of successful rapid learning of vowel-contingent rules. In *perceptual* phonotactic learning, the vowel-contingent rules are easily learned in one session (Onishi, et al., 2002). The task of perception is quite different from the task of syllabification during production. Keeping track of how vowels and consonants cue one another is important for word recognition (e.g., Pitt, Myung, & Altieri, 2007), and thus this information would not be backgrounded in perception. The second example involves testing the productions of children. Smalle, Muylle, Szmalec, and Duyck (2017) used the speech-error task and found that nine-year old children learned a second-order vowel-contingent rule on Day 1 and that the learning effect did not increase over subsequent days. (Adults tested on the same materials only showed the effect starting on the second day.) This finding provides evidence that the children’s representations of vowel–consonant conjunctions were fully available. Perhaps backgrounding them, presumably at a later age, is part of the increasing inflexibility of the production system with age.

The study presented here provided a new test of this framework, by going beyond second-order vowel contingencies. Instead, we considered the influence of suprasegmental features on phonotactic learning. These are linguistic features that are larger than a single consonant or vowel—including, for example, syllable stress and tone. A recent perception study by White, Chambers, Miller, and Jethava (2017) showed that listeners could learn phonotactics conditioned on stress cues. However, there has been no investigation of the learnability of suprasegmental-conditioned phonotactics in the language production system.

The experiments in this article investigated second-order constraint learning with two kinds of suprasegmental contexts: tone (Exp. 1) and stress (Exp. 2). *Tone* is the use of pitch to distinguish lexical items. Although English speakers use changes in pitch pragmatically, English is not a tone language, as, for example, Chinese languages are; in English, different intonation patterns on the same segment sequence do not create different words. For example, “yes!” is the same lexical item as “yes?.” *Stress*, on the other hand, is the relative emphasis on a certain syllable in a word. In English, different stress can create different words—for example, “INsight” and “inCITE” (capital letters indicate stress) even though, arguably, they have the same segment sequence. From the perspective of our theoretical framework, the crucial difference between tone and stress is that stress, but not tone, would be expected to be an input to the syllabification process. We developed these predictions first for the tone experiment.

English’s use of intonation patterns includes two functions that, when applied to single-syllable utterances, create analogues to the lexical tones used, for example, in Mandarin Chinese. In an utterance such as “yes?,” the rising pitch pattern is quite similar to the Mandarin second tone, and in an utterance such as “yes!,” the falling pattern corresponds to the Mandarin fourth tone. We will use the distinctive rising (?) and falling (!) pitch patterns as “tones” in our study, and refer to them as such, while recognizing that English intonation patterns are not technically lexical tones.

The framework for phonotactic learning in production that we summarized above predicts that a tone-contingent second-order constraint—in which, for example, /f/ is an onset and /b/ is a coda if the tone is rising, but /b/ is an onset and /f/ is a coda if the tone is falling—should not be learnable by adult English speakers. That is, English “tone” should be like speech rate. The framework assumes that the input to the syllabification process is restricted by the features that distinguish lexical items, and English pragmatic tone is not such a feature. We recognize that at some point during linguistic development, tone must have had the potential to be such a feature. Lexical tone is a feature of many languages, and moreover, different tones can be associated with different phonotactic patterns (e.g., in Cantonese, the segmental content of the coda relates to which tones are possible on a syllable; Zee, 1999). By some later point, however, English speakers have learned that pitch does not distinguish lexical items and, hence, is not considered an input to the lexically relevant aspect of phonological encoding. We also recognize that the speech production system must have mechanisms for applying intonation patterns to planned utterances. The proposal from the framework, though, is that this process is external to the lexically relevant phonological encoding step. Of course, this assumption may be incorrect, with intonational features being internal to the step. If so, the expectation is that a second-order tone rule would ultimately be learnable.

In Experiment 1, the tone experiment, we used the same experimental design that had been used to demonstrate learning of second-order constraints such as this one: /*f*/ *must be an onset and* /*s*/ *must be a coda when the vowel is* /æ/, *and the reverse when the vowel is* /*ɪ*/*.* As we noted above, the many tests of this kind of constraint have invariably demonstrated learning, but only on the second testing day (Anderson & Dell, 2018). In each of these studies, the four syllables in each sequence had the same vowel. But the particular vowel used in each sequence alternated by trial. For example, on one trial, the sequence to be spoken could be “fang nas hak mag,” and on the next trial, the sequence could be “sik him gif ning.” (Note that the locations of the restricted consonants /f/ and /s/ respect the example constraint given above.) It is a simple matter to use this design to test a tone constraint: For example, /*f*/ *must be an onset and* /*s*/ *must be a coda when the tone is rising, and the reverse when the tone is falling*. Instead of alternating trials of two different vowel sequences, we alternate trials in which all four syllables are said with a rising tone marked by “?” after each syllable, and trials in which all syllables have a falling tone, marked by “!.” English speakers naturally use a rising tone to say, for example, “mef?,” and a falling tone to say, for example, “mef!” Thus, in both the vowel contingency studies and the new tone study, the conditioning factor (vowel or tone) was marked by a single orthographic symbol for each syllable. Aside from changing the contingency from the vowel to the tone, the only other significant design change was that we attempted to increase experimental power. The most powerful of the vowel contingency studies tested a total of 12 participants each doing 96 trials per day (see Anderson & Dell, 2018). Overall, an experiment of this size records, on each day, 13,824 attempts to produce a syllable. We increased the number of participants to 18, and hence a total of 20,736 syllables were attempted each day.

## Experiment 1: Tone

### Methods

#### Participants

A total of 18 native monolingual English speaking students (13 females, five males) from the University of Illinois at Urbana-Champaign provided the data. These 18 participants were part of a larger group of 23, all of whom underwent pretesting as described below, which was carried out to ensure that all analyzed participants accurately and consistently produced the tone patterns in the artificial materials. The 18 successful participants were paid $20 for participating in one session per day for two separate days. The participants were randomly assigned to one of six constraints, described below. The use of these six constraints ensured that each consonant had an equal chance of participating in the experimental constraint, and that the resulting constraints would balance consonants across rising and falling tones and onset–coda combinations.

#### Materials and procedures

Each sequence in the tone experiment consisted of four CVC syllables using the following eight consonants, /h, ŋ, f, b, k, d, m, n/, and the short e vowel, spelled with “e” and pronounced as /ɛ/. The eight consonants each appeared once in each sequence (four times as onsets and four times as codas). The actual syllables in each sequence and their order were randomly determined, except for the influence of language-wide and experiment-wide constraints: The /h/ must appear as an onset and the /ŋ/ as a coda, as dictated by English phonotactics. In addition, two other consonants followed a tone-based second-order experiment-wide constraint, and this constraint varied among participants. In the sequence, each syllable was presented as a single word—separated by a space from its neighbor syllables—and its desired tone was marked with either “?” or “!” after the syllable. Participants were randomly assigned one of six constraints: feb!–bef?, bef!–feb?, dek!–ked?, ked!–dek?, men!–nem?, nem!–men? This notation stands for what tone-based constraints participants received. For example, feb!–bef? means the constraint, /*f*/ *must be an onset and* /*b*/ *must be a coda if the tone is falling, but* /*f*/ *must be a coda and* /*b*/ *must be an onset if the tone is rising*. In pilot work, we showed that English speakers naturally use a rising tone to say, for example, “bef?,” and a falling tone to say “bef!.” As in the standard design used for testing second-order phonotactic learning, all syllables of a sequence had the same tone, and “?” and “!” trials alternated. For example, two adjacent sequences in the feb!–bef? condition might be:

- hem! feb! dek! neng! (2) mef? beng? hen? dek?

Each participant performed 96 trials on each of two days. On a single trial, a sequence was visually presented on a computer screen. Participants recited the sequence following a computer-generated metronome, associating a tone with each syllable. For each sequence, participants first read it aloud once at a rate of 1.1 syllable per second, and then repeated the sequence three times without pause at a faster rate of 2.8 syllable per second. All recitations were digitally recorded. There were both practice and experimental sessions on the first day, but only an experimental session on the second day.

In the practice session, participants were shown two practice trials, one with rising tones and one with falling tones. The experimenter instructed the participants how to pronounce the syllables, and they practiced these several times until they had accurately produced the patterns and their timing with the metronome. If participants could not reproduce the tones or the metronome timing after 5 min of such training, the experimenter would remove them from the study (without penalty to the participant). The logic for excluding participants who had difficulty is that, if they could not pronounce the tones correctly, anything that they would have learned would not have been a true tone-based rule.

During the experimental session, the experimenter sat next to the participants and listened to their pronunciation of the experimental trials. The experimenter would correct participants if they produced a tone incorrectly. If the participants, despite having passed the practice session, still exhibited difficulty after 20 trials, the experimenter marked this participant as eliminated, and their data were not examined. After the 20-trial period, the experimenter would leave the room, to let the participants finish the rest of the experiment unsupervised.

Aside from the opportunity to eliminate participants because of failure to complete the practice and because of a judged lack of tone accuracy during the first 20 trials of the experiment, the experimenters reserved the right to eliminate participants as a result of an initial scrutiny of their recorded productions after the experiment was over. In all cases, though, participants were eliminated before any consideration of their consonant slips, which constituted the key data.

### Results and discussion

Consonant slips that occurred during the rapidly produced recitations were the data of interest. There were two coders, and each identified slips from high-quality audio recordings of the sessions. One coder examined the output of nine participants, and the other did the other nine participants. The coders identified all errors, without attending to the condition that the participant was in. In addition, a subset of the recordings, containing 1,152 syllables, were coded by both coders, in order to determine coding reliability. In this reliability check, both coders agreed that there were no errors in 1,752 out of the 2,304 consonants, and agreed on the nature and presence of 508 errors. The overall agreement between the two coders was 98.1%. This reliability is comparable to those in previous speech error studies (e.g., Dell et al., 2000; Warker & Dell, 2006). In addition to finding slips, the coders also judged whether the tone of each syllable was produced correctly as rising or falling. If a particular syllable’s tone was not correct, no segmental errors were counted from that syllable. The tones were generally produced accurately; only 0.4% of rising-tone syllables were eliminated, and only 0.8% of falling-tone syllables were eliminated.

Speech errors involving experiment-restricted (hereafter referred to simply as “restricted”) and unrestricted consonants occurred approximately equally on rising-tone (2,136 errors) and falling-tone (2,336) syllables. The speech errors involving consonants restricted by experiment-wide constraints were classified as legal if they followed their respective constraints (e.g., in a rising tone sequence, onset /f/ slipping to coda position for participants in the feb!–bef? condition), and illegal if they did not. The slips involving unrestricted consonants constituted the baseline. A slip of an unrestricted consonant was classified as legal or not as in previous studies, that is, by examining whether the slipping consonant preserved or changed its syllable position. For example, if the original position of the consonant was as an onset, and it slipped to another onset position, the slip would be legal, and if it slipped to a coda position it would be illegal.

The data analysis procedures followed standards that had been used for other second-order phonotactic-learning studies that involved two testing sessions (e.g., Anderson & Dell, 2018; Warker et al., 2008). Specifically, we tested three planned contrasts: between restricted and unrestricted legality on Day 1, restricted and unrestricted legality on Day 2, and the interaction contrast between day and restrictedness. Prior studies have established that the proper directional null hypothesis for the pairwise contrasts is that the legality of restricted condition is not greater than the legality of the unrestricted baseline, and for the interaction that excess legality of the restricted condition on Day 2 is not greater than the excess legality of that condition on Day 1. As in previous studies using these methods, the nonparametric Wilcoxon signed-rank test was used to evaluate the contrasts, because the distribution of the relevant error rates per participant is quite variable.

**Fig. 1:**
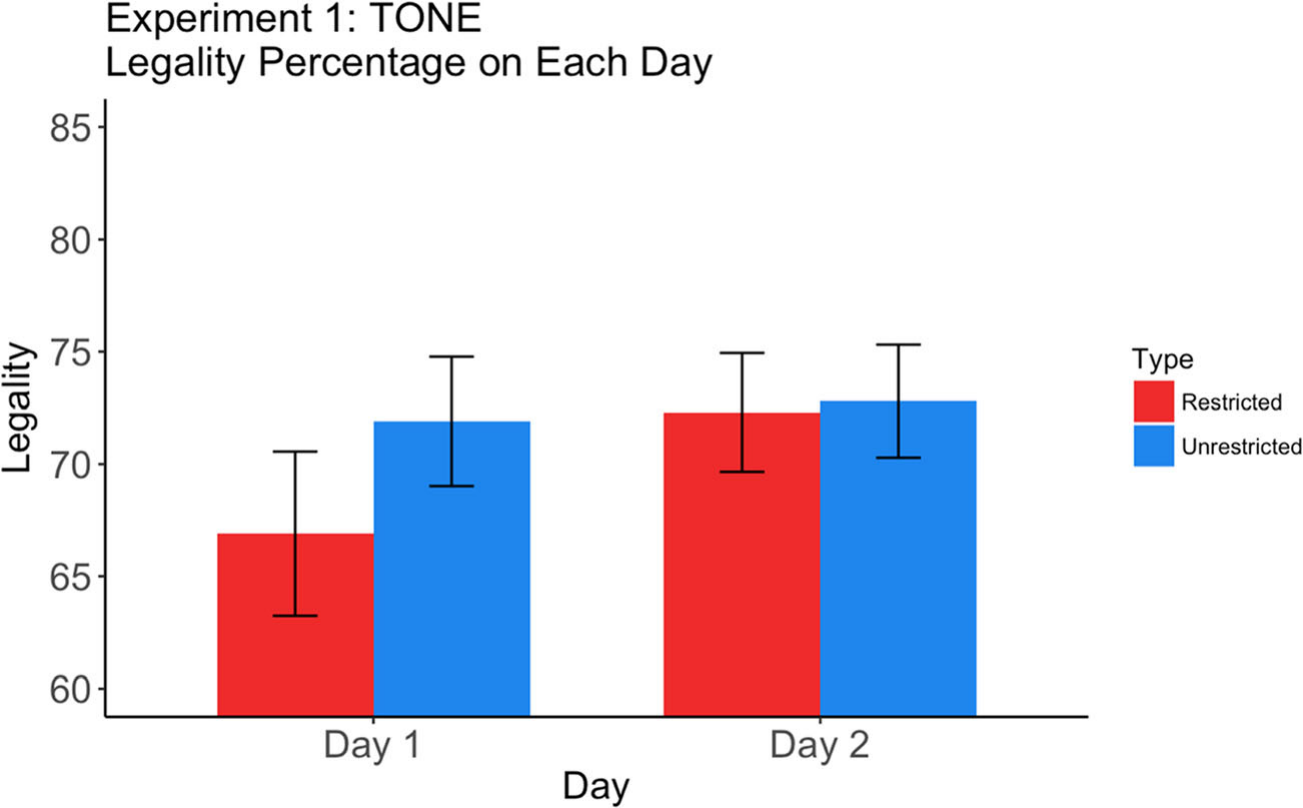
Legality of restricted and unrestricted slips for the tone experiment, as a function of day.

There were no significant differences between the restricted and unrestricted legality proportions on either Day 1 (5% difference in the wrong direction) or Day 2 (0.5% difference in the wrong direction), as well as no significant interaction (Wilcoxon *p* = .065). See Table 1 and Fig. 1. The level of “legality” of the unrestricted slips (around 70%) was consistent with all prior studies that have used this kind of design to test for the learning of second-order phonotactic constraints (e.g., Warker, 2013). But there was clearly no excess legality for the restricted slips. These results reveal no learning of tone constraints on either day, suggesting that tone, at least as it was implemented here, cannot serve as a conditioning factor for English speakers acquiring new phonotactic constraints on consonant placement.

**Table 1:** Percentages of legal errors of unrestricted and restricted consonants in Experiment 1 (tone)

	Unrestricted	*SE*	Restricted	*SE*
Day 1	71.9	2.9	66.9	3.7
Day 2	72.8	2.5	72.3	2.6
Total errors (count)	2,901		1,751

There are two issues that should be addressed, though. Is this a convincing null result? And if it is, is this simply because suprasegmental features generally do not work well as conditioning factors in these kinds of experiments? The first question is easy to address: The study had more power than all of the previous successful studies involving the learning of second-order constraints in production. As a result of this increase in power, over 4,000 relevant slips contributed to the data (Table 1). From the previous studies, we know that if a positive result could be obtained, it would have occurred on Day 2. Yet the restricted and unrestricted proportions were nearly identical on that day. One way to quantify the support for the null hypothesis is to compare the likelihood of the data given the null hypothesis to the likelihood of the data given an alternative hypothesis (the Bayes factor; e.g., Jakobsen, Gluud, Winkel, Lange, & Wetterslev, 2014). Calculating Bayes factors requires making many assumptions, but perhaps the simplest approach is to compare a point null hypothesis with point alternatives determined from other research, and to assume identical priors on each alternative and the null. If we set the alternative hypothesis as the mean second-order Day 2 effect (restricted – unrestricted legality) obtained from Anderson and Dell’s (2018) meta-analysis (14%), the null hypothesis is favored over the alternative by 3,155 to 1. More conservatively, if we assume that the alternative is the smallest obtained second-order effect (7%), the data still provide strong support for the null, by a factor of 9 to 1. Given this, we are reasonably confident that the second-order tone contingency is not learnable by the methods that have invariably demonstrated learning of a second-order vowel contingency. The tone study used exactly the same design as the previous vowel studies, only with more participants, and the null hypothesis was a far more defensible characterization of the data than the alternative hypotheses derived from the results of the vowel studies.

To address the question of whether suprasegmental factors in general do not make effective cues in these kinds of studies, we next considered the factor of stress.

## Experiment 2: Stress

Here we tested constraints such as this one: /f/ must be an onset and /ʃ/ must be a coda in stressed syllables, but /ʃ/ must be an onset and /f/ must be a coda in unstressed syllables. These constraints have the same form as the second-order vowel-contingent constraints that were learnable after the first day, as well as the same form as the tone constraints that were not learnable. From the perspective of the framework that we summarized earlier, we would expect the stress constraint to be learnable, because stress distinguishes English words and, hence, would be an input to phonological encoding. Stress interacts with syllable structure in English in a number of ways (see, e.g., Hayes, 1995). There is the weight-to-stress principle, which proposes that syllables with long vowels or complex codas tend to be stressed. Stress affects both vowels (vowel reduction in unstressed syllables) and consonants (e.g., /*t*/ is flapped if it occurs between a stressed and unstressed syllable). At the same time, though, the status of a syllable as stressed or not is not a useful cue for whether a particular consonant may be restricted to the onset or coda position (as /h/ and /ŋ/ are). In this respect, stress is like a syllable’s vowel. It is an important aspect of English word form, but it provides little information about how consonants may be restricted to onset or coda position. Thus, within our framework, we expected the stress constraint to be learnable in the same way that a vowel-contingent constraint is; the learning effect should appear on the second testing day, because of the need for a consolidation period to restore the functionality of representations that can code for the conjunction of stress values and consonants (e.g., /f/ in the context of a stressed syllable).

### Method

The stress experiment kept its methods as close as possible to those of the tone experiment, while ensuring that the stress manipulation effectively manipulated stress as experienced in English. The stress constraints to be learned were directly analogous to the tone ones: For instance, /*f*/ *must be an onset and* /*ʃ*/ *must be a coda if a syllable is stressed, and* /*ʃ*/ *must be a coda and* /*f*/ *must be an onset if a syllable is unstressed*. As before, each sequence had exactly four CVC syllables with eight distinct consonants and a single repeating vowel. Likewise, there are 96 such trials on each of two days. The only major difference from the tone study arose from two facts: (1) English avoids stress clash—that is, adjacent stressed syllables within a phonological domain are avoided; (2) English tends toward stress-timing—that is, stressed syllables are produced at approximately regular intervals. Thus, our stressed and unstressed syllables alternated within a sequence, and stressed but not unstressed syllables aligned with metronome signals.

#### Participants

A total of 18 native monolingual English speaking students (ten females, eight males) from the same population as before provided the data. These 18 participants were part of a larger group of 48, all of whom were pretested as in the tone experiment. The 18 successful participants were paid $20 for participating in one session per day for two separate days. The participants were randomly assigned to one of six constraints, which counterbalanced the materials and conditions: FISH–shif, SHIF–fish, DIK–kid, KID–dik, MIN–nim, and NIM–min (as we describe below).

#### Materials and procedures

The sequences were randomly generated as before, subject to language-wide and experiment-wide constraints. Each sequence contained eight consonants /h, ŋ, f, ʃ, k, d, m, n/, each of which appeared only once per sequence, and orthographic “i” (/ɪ/) was the vowel used in all syllables. Notice that there are two changes from the phonemes used in the tone experiment. First, the /b/ used in the tone experiment was replaced with /ʃ/ (spelled “sh”). This change made the consonants more distinct from one another, to aid coding of the often difficult-to-hear consonants in unstressed syllables. Second, the short “i” vowel was chosen instead of the short “e” vowel used in the tone study, because it can occur in both stressed and unstressed syllables of English. We acknowledge that the phonetics of stressed and unstressed utterances of “i” vowels in the study would not be exactly the same, but we assume that their differences would be those determined by stress rather than by different lexical representations.

The first two, and the last two, syllables of each sequence were each concatenated as a single two-syllable “word” (spelled without a space between the syllables). Half of the sequences were made up of two trochaic (stress on the first syllable) words, and half had two iambic (stress on the second syllable) words. These two sequence types alternated. The use of both trochaic and iambic trials ensured that the stress constraints present in the materials concerned the stress value of the syllable and not its word position. The sets of sequences prepared for each participant exhibited one of the six possible constraints mentioned above. The FISH–shif constraint was shorthand for the rule that /*f*/ *must be an onset and* /*ʃ*/ *must be a coda if a syllable is stressed;* /*f*/ *must be a coda and* /*ʃ*/ *must be an onset if a syllable is unstressed*. The other five constraints were defined analogously. An example of two consecutive trials in the FISH–shif condition would be (1) HIMdin FINGshik; (2) nifMISH kidHIN (with capital letters indicated stressed syllable). Language-restricted, experiment-restricted, and unrestricted consonants were defined as in the tone study, the only difference being that the constraints were stress-based instead of tone-based.

As before, each participant received 96 trials on each day. The sequences were presented visually, one at a time, to the participants on the computer screen, with word boundaries marked with spaces and stressed syllables capitalized. Participants recited the sequence following the tone of the metronome, associating a tone with a stressed syllable. For each sequence, participants first read it aloud once at a rate of 1.0 word per second, and then repeated the sequence three times without pause at a faster rate of 1.9 words per second. All recitations were digitally recorded. Practice sessions were conducted as before in the tone experiment, and participants were replaced using the same procedure. More participants were replaced in the stress than in the tone study, likely because accurately producing stress timing using unknown words does not come easy to many participants. Because such timing is a central feature of English stress, we felt it necessary to require it of the participants’ speech.

### Results and discussion

The errors that occurred in Experiment 2 were transcribed and coded as before. In the reliability check, two coders coded the same set of 1,152 spoken syllables (2,304 consonants). Both coders agreed that there were no errors on 1,882 out of the 2,304 consonants, and agreed on the nature and presence of 383 errors. The overall agreement between the coders was 98.3%. If a word was given the wrong stress pattern, no segmental errors were counted from either syllable of the word. These stress errors occurred on 2.7% of the trochaic words and 3.1% of the iambic words. Thus, these participants were producing the stress patterns as directed. Please note that these elimination percentages are somewhat higher for the stress experiment than for the tone experiment, consistent with our claim that the stress procedure, which necessitates alternations of stressed and unstressed syllables, was more difficult.

There were 1,225 errors (702 on Day 1, 523 on Day 2) involving experiment-restricted consonants (restricted errors). These were classified as legal if the slips still conformed to the experimental constraint assigned to that participant. For example, when a participant said “FISHmik HINGnid” when he or she was supposed to say “FIMshik HINGnid” in the FISH–shif condition (i.e., /f/ is onset and /ʃ/ is a coda if the syllable is stressed, and vice versa if the syllable is not stressed), the slip of the experiment-restricted /ʃ/ was considered legal, since /ʃ/ was supposed to be a coda in stressed syllables. But if the participant said “FIKmish HINGnid,” then the slip was illegal, since /ʃ/ should not be a coda in unstressed syllables. We used the 2,447 errors involving unrestricted consonants (or unrestricted errors) as the baseline. As before, we note that the unrestricted consonant involved in each error was “restricted” within the sequence, because each consonant occurred exactly once in every sequence. Thus, we can label the potential positions that it could slip to within this sequence as “legal” or “illegal,” just as if the consonant had been restricted, and in so doing classify the slip as legal or illegal. For example, if /m/ were unrestricted in the trial “FISHmik HINGnid” in the FISH–shif condition, we can note that /m/, in this sequence, was restricted the same way that /ʃ/ was restricted for the entire experiment (i.e., it is an onset in stressed syllables and a coda in unstressed syllables). Then the proportion of unrestricted errors that were legal in this sense is an unbiased baseline to compare to the legality of restricted slips.

We expected no difference between the restricted and unrestricted legality proportions on Day 1, since previous studies on second-order learning have shown that any such learning only shows up after sleep. But we expected restricted legality to be significantly higher than unrestricted legality on Day 2. As before, we tested the three planned contrasts with nonparametric methods: between restricted and unrestricted legality on Day 1, restricted and unrestricted legality on Day 2, and the interaction between day and restrictedness.

**Fig. 2:**
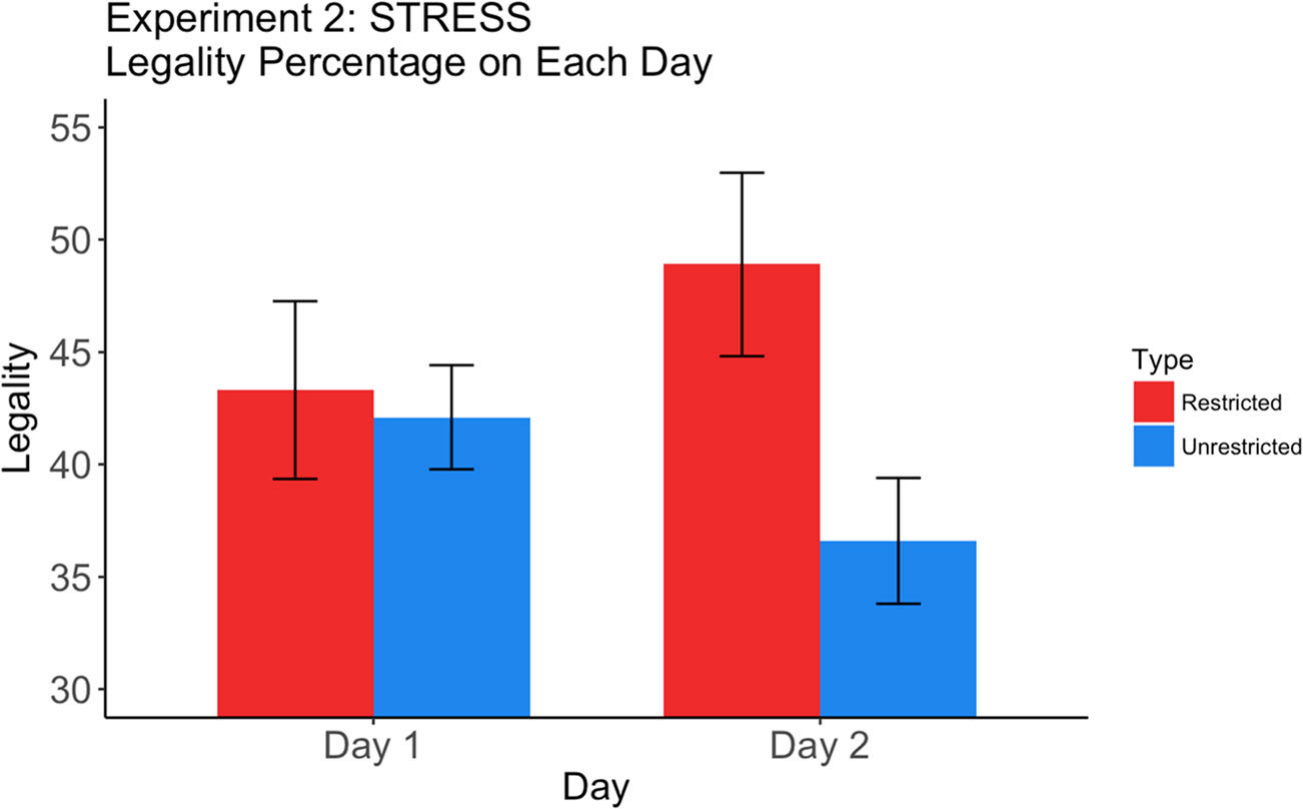
Legality of restricted and unrestricted slips for the stress experiment, as a function of day.

The results are presented in Fig. 2 and Table 2. We found no evidence of learning on Day 1 (Wilcoxon *p* = .17), but a robust 12.3% effect on Day 2 (Wilcoxon *p* < .05). In addition, the interaction between days and restrictedness was significant (Wilcoxon *p* < .05). The results were exactly as predicted. They supported the hypothesis that English speakers can learn stress-based constraints, but not until Day 2. The magnitude of the difference on Day 2 is close to the mean expected effect for other second-order effects (14%; Anderson & Dell, 2018). We mention one caveat, though, that specifically impacts the interaction contrast: There was a drop in the baseline (unrestricted legality) from Day 1 to Day 2, a drop that contributed to the interaction. Baseline effects may vary as a function of testing day, and consequently, it is best to compare restricted and unrestricted slips on the same day rather than across days, which is effectively what our contrasts do.

**Table 2:** Percentages of legal errors of unrestricted and restricted consonants in Experiment 2 (stress)

	Unrestricted	*SE*	Restricted	*SE*
Day 1	42.1	2.3	43.3	4.0
Day 2	36.6	2.8	48.9	4.1
Total errors (count)	2,447		1,225	

Another notable feature of the stress results was the tendency for slips to be more common on unstressed (2,139 slips) than on stressed (1,533 slips) syllables. This was expected, from findings that attended or otherwise emphasized syllables are relatively immune to production errors (Nozari & Dell, 2012). Importantly, the effect of learning—the higher legality of restricted than of unrestricted slips on Day 2—was numerically present for errors in both stressed (8% effect) and unstressed (15% effect) syllables. These percentage differences fall within the range of other observed second-order effect sizes (Anderson & Dell, 2018).

One noticeable difference between the results of the tone and stress experiments is that the legality of both restricted and unrestricted slips is considerably higher in the tone experiment than in the stress experiment. This can be fully explained by the syllable-position effect on speech errors—the fact that onsets generally slip to onset positions and codas generally slip to coda positions. In the tone experiment, all of the syllables in a trial had the same tone, and therefore the legal slips of a consonant would necessarily be at the same syllable position (e.g., the only allowed legal position for an onset /f/ was at the onset). In the stress experiment, there were both stressed and unstressed syllables in a trial, and hence the legal slip of a particular consonant could be to either onset or coda positions. Because of these facts, legal slips in the stress experiment often had to violate the syllable position effect (e.g., an onset moves to a coda position, or vice versa), whereas the ones in the tone experiment did not. Thus, the syllable position effect worked against the stress-based rule. In the tone experiment, the syllable-position effect worked along with the tone-based rule.

## General discussion

The results were as predicted. Experiment 2 showed that English speakers can learn stress-based phonotactic constraints, but not until the second day. Previous phonotactic learning studies involving production have also found this phenomenon of delayed learning of a constraint in which some linguistic factor (e.g., the vowel) constrained whether a consonant can be an onset or a coda (Warker & Dell, 2006). Experiment 1 showed that English speakers could not learn analogous tone-based constraints after two days of training. In this respect, for these speakers, tone is like speech rate (Warker et al., 2008).

We had predicted these results from a framework in which speech errors arise during phonological encoding and specifically during the assignment of retrieved segmental material to syllable positions. Crucially the phonological encoding process is adaptive. It changes based on linguistic experience, both long-term experience and experience in the laboratory. Furthermore, the process is assumed to have access to information retrieved from the lexicon, which would include stress patterns, but not tones, for English speakers. Thus this system is capable of learning how stress might constrain the location of consonants. Tone as used by English speakers, though, is not an input to this syllabification process and thus comparable constraints based on tone could not be learned. Finally, the fact that a second-order stress constraint only appears to be learned after a consolidation period is attributed to the system’s knowledge that stress in English is not a strong cue for whether a particular consonant is an onset or coda. The same is true for vowels. Thus, it is assumed that the resources that can represent these kinds of second-order contingencies (e.g., in the context of the model, hidden units that represent conjunctions of stress values or vowels, and particular consonants) are, in some way, unable to operate immediately. That is, they are backgrounded. The framework does not specify a mechanism for such backgrounding.

Furthermore, our findings may relate to two phenomena: one well known, one less so. The well-known fact is the ubiquity of foreign accents. Older speakers usually have great difficulty learning to produce phonological contrasts that are not present in their native language; for instance, producing and recognizing lexical tone is quite difficult for adult English speakers (e.g., Shen, 1989). We see our findings as relevant for such phonological critical-period effects. But we note that there is a unique aspect to our findings: The inability to learn the tone-based constraint occurred even though our speakers could, for the most part, *fully produce* all of the syllable-tone combinations that they experienced. Their failure to learn was not a sensory or motor problem. The participants’ only failure, as it were, was that their slips did not obey rules such as that /f/ is an onset when the tone is rising, but a coda when the tone is falling. Thus, our findings speak to a role of linguistic experience as defining the abstract form of what is learnable, not the particular sensory–motor features and feature combinations.

The other prior results that relate to our findings concern discoveries about how the properties of one’s language impinge on experimental studies in the perception of statistical and artificial grammar learning. Seidl, Cristia, Bernard, and Onishi (2009) demonstrated that English and French learning infants could learn a new phonotactic constraint based on vowel nasality. Slightly older English learning infants could not learn the constraint, perhaps because they had learned that nasality is not phonemic in English vowels. Also, the ability of participants to detect recurring syllable sequences in the classic word segmentation paradigm (e.g., Saffran, Aslin, & Newport, 1996) is impacted by information from one’s native language, both native phonotactics (Finn & Hudson Kam, 2008) and native syllable-syllable association strengths (Siegelman, Bogaerts, Elazar, Arciuli, & Frost, 2018).

We wish to emphasize that we have not proven that the contrast between our findings with stress and with tone is specifically due to linguistic experience. Perhaps there is some other reason why tone rules are difficult to learn. At this point, all we can say is that our hypotheses, which were derived from the differences in how stress and tone work in English, were supported. We can, though, generate clear predictions for future research. If analogous studies of suprasegmental learning could be conducted on tone languages that have some diversity of coda consonants differing results are expected. For example, Cantonese, like other Chinese languages, has lexical tone but no lexical stress. Moreover, tone interacts with phonotactics in the coda position. Thus, it is the opposite of English and opposite results would be expected if analogous studies could be constructed.

In addition, important developmental questions arise from our findings that can easily be addressed. For example, as we mentioned, Smalle et al. (2017) showed that 9-year-old children can be tested in the phonotactic-learning paradigm that we used here, and that they can learn second-order vowel-contingent constraints without a consolidation period. The obvious question from our research is whether such children (here, Dutch children who do not know a tone language) would learn the tone-based constraints.

The big picture is that our study, together with the other studies of second-order phonotactic learning in production, are informative about both consolidation effects (the need for sleep in learning the stress rule) and age of acquisition/critical periods (the unlearnability of tone by English-speaking adults). At present, no learning theory simultaneously addresses both of these important questions (see Smalle, Page, Duyck, Edwards, & Szmalec, 2018), largely because no data sources show both clear sleep dependencies and variation in learnability that could be due to age and experience. The present studies take a small, but important step, toward providing such data.

In conclusion, through the two experiments in this article, we investigated the limits of context-dependent phonotactic constraint learning, and the results showed that English speakers could learn stress-based but not tone-based phonotactics in our experiments. This suggests that learning of English shapes the adult production system in such a way that stress and tone features are differentially available to phonological encoding, thus subsequently enabling the learning of new stress-based but not tone-based constraints.

## Footnotes

1 The legality of unrestricted slips is considerably greater than 50% because of the syllable-position effect (Nooteboom, 1973), which is a statistical tendency for onsets to move to onset positions and codas to move to coda position in slips. The syllable-position effect could contribute in part to the high levels of legality for the language-restricted and experiment-restricted slips. Hence, the baseline condition needs to reflect this factor as well to create an unbiased comparison.

2 In the phonotactics of English, one sees 2nd order structures in allowable onsets—for example if the onset begins with /s/, it can be followed by /n/, but not by /r/; if the onset begins with /d/, the reverse is true.
